# Effects of bioformulation prototype and bioactive extracts from *Agaricus bisporus* spent mushroom substrate on controlling *Rhizoctonia solani* of *Lactuca sativa* L

**DOI:** 10.3389/fpls.2024.1466956

**Published:** 2024-10-24

**Authors:** Rudy Cignola, Gaia Carminati, Andrea Natolino, Alessandra Di Francesco

**Affiliations:** Department of Agriculture, Food, Environmental and Animal Sciences, University of Udine, Udine, Italy

**Keywords:** soil-borne disease, *Aureobasidium pullulans*, spent mushroom substrates, formulation, plant growth promotion

## Abstract

**Introduction:**

Food security and waste management represent the main challenges that need to be addressed in the near future. The use of bioformulations and bioactive compounds obtained from agricultural wastes could represent some of the solutions for the management of soil-borne pathogens.

**Methods:**

In the present study, *Aureobasidium pullulans* strain AP1, tested in oil dispersion (OD) formulation prototype and bio-extracts [hot water extract (HWE) and warm water extract (WWE)] derived from spent mushroom substrate (SMS) of *Agaricus bisporus*, was tested as sustainable strategies to manage *Rhizoctonia solani* of lettuce.

**Results:**

By *in vitro* assays, AP1OD at 600 mg L^−1^ displayed an inhibition by 57% of pathogen mycelial growth, and the SMS extract WWE (40°C) showed a growth stimulation of lettuce seedling by 27%. By *In vivo* assays, AP1OD formulation used against *R. solani* reduced by 66.6% the soil-borne pathogen incidence on lettuce plants, and both bio-extracts significantly stimulated lettuce leaves and roots growth (>200%). AP1OD formulation and HWE treatments increased the lettuce genes expression levels (*ggps* and *hppd pdx1*) mainly imputed to plant antioxidant potential, vitamin E, and vitamin B6 biosynthesis.

**Discussion:**

The present study reported the potential of a new formulation and two bio-extracts, derived from an agricultural waste, to use against *R. solani* of lettuce, respectively, with antifungal and biostimulant properties.

## 1 Introduction

Lettuce (*Lactuca sativa* L.) is one of the most globally cultivated and consumed vegetable (Mou, 2008). Within *Lactuca* genus, hundreds of species and varieties offer different nutritional elements, making lettuce a versatile and nutrient-rich choice (Křístková et al., 2008; Kim et al., 2016). Over 27 million tons are produced annually (FAO, 2021), making of lettuce the most important cultivated plant in the group of leafy vegetables (Křístková et al., 2008).

Nevertheless, different soil-borne pathogens cause critical damages to horticultural crops and in particular *Rhizoctonia solani*. The pathogen is known to cause a wide range of economically relevant plant diseases, which include brown patch, damping off in seedlings, root rot, and belly rot of lettuce (Erlacher et al., 2014). Over the last decades, new strategies such as the biocontrol were investigated to manage this pathogen also in lettuce, due to the elevated demand of organic productions (Aggeli et al., 2020). The global population presents imminent challenges in terms of both food security and waste management in the near future; so due to the increasing demand of organic food, the biocontrol strategies, such as the use of biocontrol agents (BCAs) or bioactive compounds obtained from agricultural wastes, could represent some solutions for the management of soil-borne pathogens (El-Tarabily, 2004; Di Francesco et al., 2021). Therefore, the use of plant growth promoters (PGPs), such as microorganisms, could be the key to maintain high levels of crops productivity. In fact, PGP microorganisms, in particular yeasts, are able to colonize plant parts and contribute to the modulation of the host phytohormones production, improve soil fertility, increase nutrient availability, enhance resistance to abiotic stress, and inhibit different pathogens (Nimsi et al., 2023). *Aureobasidium pullulans*, a ubiquitous yeast, is able to produce indole-3-acetic acid, an auxin class hormone responsible for the initiation and elongation of roots and stems and for the development of lateral roots and root hairs, which are essential conditions for nutrients’ uptake, plant growth promotion, and stress resistance (Ignatova et al., 2015). Concerning the use of BCAs, *Trichoderma* spp. have proven to be effective against numerous soil-borne pathogens, such as *Pythium* spp., *Rhizoctonia* spp., *Fusarium* spp., and *Sclerotinia* spp (Woo et al., 2023). Several products have been developed and commercialized for the application against those pathogens, mainly based on fungi and bacteria. Unfortunately, yeasts have received less attention with respect to fungi and bacteria as soil-borne pathogens BCAs, although they play a fundamental role in the soil niches (Palmieri et al., 2022). About the agricultural wastes, every year, tons of spent mushroom substrates (SMSs) derived from the cultivation of edible fungi are generated, creating an important ecological problem (Atallah et al., 2021; Martín et al., 2023). Given the high number of bioactive compounds contained in the SMS, exploring new ways to re-use it could have both an environmental and economical substantial impact. In particular, adopting sustainable methods like water extraction could yield benefits (Martín et al., 2023).

Based on these considerations, the aims of the present work were to: *i*) evaluate the biocontrol ability of *A. pullulans* experimental formulation against *R. solani* by *in vitro* and *in vivo* assays; *ii*) study the growth promotion effect exerted by *Agaricus bisporus* SMS extracts and *A. pullulans* formulation on lettuce plants (leaf, root, and stem); and *iii*) verify the expression levels of genes correlated to the antioxidant potential and vitamin E and B6 biosynthesis, induced by both treatments on lettuce plants.

## 2 Materials and methods

### 2.1 Fungal pathogen and plant

*Rhizoctonia solani* strain Riz4 belonged to the mycological collection of Department of Agriculture, Food, Environmental and Animal Sciences of Udine University. The pathogen was originally isolated from symptomatic lettuce plants cv. “Lollo bionda.” The pathogen was grown on Potato Dextrose Agar (PDA; 39 g L^−1^ of distilled water, Oxoid, UK) for 5 days at 20°C. Mycelial plugs of 6-mm diameter of 5-day-old colony were used for the *in vitro* assays. For the *in vivo* assay, 20 fungal mycelial plugs were placed in 2-L flask containing 500 mL of Malt Extract Broth (30 g L^−1^ of distilled water, Oxoid, UK) and incubated at 20°C on a rotary shaker (200 rpm) for 5 days. The culture was then centrifuged at 5000×*g* for 20 min at 4°C, and the mycelium was washed with distilled water, filtered through two sterile miracloth layers, and homogenized by using a mixer.

*Lactuca sativa* plants were obtained from organic seeds cv. “Lollo bionda” (Blumen Group, Italy) seeded in 50-mL pots filled with moss, until the fifth expanded leaf (18 days), under daylight conditions at 20 ± 2°C and 70% of relative humidity.

### 2.2 Yeast formulation and spent mushroom substrate extracts

*Aureobasidium pullulans* strain AP1 that belong to the mycological collection of Udine University was used as active substance of the prototype bioformulation AP1OD, developed in collaboration with Clever Bioscience s.r.l. (Campospinoso, Italy) (Cignola et al., 2024). The yeast strain was formulated as oil dispersion (OD) with the concentration of the viable active ingredient set to 1 × 10^7^ cells g^−1^. The formulation was prepared with food grade and eco-friendly surfactants provided by the Company (composition not disclosable) (Cignola et al., 2024).

For the bio-extracts, SMS of *Agaricus bisporus*, provided by “Consorzio Funghi Treviso” (TV, Italy) in summer 2023, was blended with sterile water at 1:3 (w/v) ratio and subjected to different extraction processes: hot water (HW at 90°C) and warm water (WW at 40°C). The aqueous part was filtered, centrifuged at 4,000 rpm for 15 min, and filtered through two layers of sterile cloth and stored at −20°C until the use. Bio-extracts were respectively named HWE and WWE.

### 2.2 Chemical analysis of spent mushroom substrate of *Agaricus bisporus* extracts

#### 2.2.1 Samples preparation

Five milliliters of *A. bisporus* SMS extracts derived from the HW and WW extraction methods, earlier described, were mixed with 20 mL of ethanol 96% (v/v). The mixtures were refrigerated for 24 h and used for the analysis below described.

#### 2.2.2 Reducing sugar content

The reducing sugars were determined on the supernatant fraction obtained from the ethanolic precipitation step. The Fehling’s solution reaction was prepared by mixing: 10 mL of solution A (70 g L^−1^ copper sulfate solution), 10 mL of solution B (346 g L^−1^ sodium-potassium tartrate solution in 100 g L^−1^ sodium hydroxide), and 60 mL of deionized water. A reaction mixture was prepared by adding 200 µL of each sample to 5 mL of Fehling’s solution, heated at 100°C for 30 min, and cooled at room temperature before spectrophotometer measurement. The absorbance was read at 694 nm using a UV-Vis spectrophotometer (Shimadzu UV 1650, Tokyo, Japan) compared to deionized water. A calibration curve was prepared with several standard solutions of D-(+)-glucose in the concentration range of 0–25 g L^−1^.

#### 2.2.3 Protein content

A reaction solution was prepared by mixing: 1 mL of sodium carbonate solution (2% w/v, in NaOH 0.1 N), 1 mL of copper sulfate solution, and 100 mL of sodium-tartrate solution (1% w/v). Two milliliters of the reaction solution was mixed with 400 µL of each sample and shaken, and, after 10 min, 200 µL of Folin-Ciocalteau reagent was added. After 30 min, the absorbance was read at 750 nm using a UV-Vis spectrophotometer (Shimadzu UV 1650, Tokyo, Japan) and compared to deionized water. A calibration curve was prepared with several standard solutions of bovine serum albumin in the concentration range of 0–800 mg L^−1^.

#### 2.2.4 Polysaccharides content

Polysaccharides were determined by Size Exclusion High-Performance Liquid Chromatography (SE-HPLC). A preliminary protein denaturation step was performed by mixing 2 mL of decolored liquid extracts and 200 µL of trichloroacetic acid solution (20% v/v). After 30 min of reaction, the samples were centrifuged, and the supernatant was recovered. Polysaccharides were precipitated by adding 4 mL of ethanol 96% (v/v). The precipitated pellet was separated by centrifugation, washed twice with ethanol (96% v/v), suspended in 2 mL of MilliQ water, and filtered by 0.22-µm cellulose acetate membrane before injection. SE-HPLC separation was achieved using a binary pump Model LC 250 (Perkin-Elmer, Waltham, MA, USA), equipped with a manual injection valve (type 7125 NS Rheodyne, Rohnert Park, CA, USA) and a refractive index detector RID-10A (Shimadzu, Kyoto, Japan). The column was an Ultrahydrogel 250 (6 µm, 300 mm × 7.8 mm, Waters, Milford, MA, USA). The mobile phase was MilliQ water, and the separation was performed in isocratic conditions, with a flow rate of 0.7 mL min^−1^; injection volume was 20 µL. Total polysaccharides were quantified by a calibration curve prepared with mannan (10–1,000 mg L^−1^).

### 2.3 *In vitro* assay: efficacy of AP1OD formulation against *R. solani* mycelial growth

Different concentrations of AP1OD formulation (0 mg L^−1^, 50 mg L^−1^, 100 mg L^−1^, 200 mg L^−1^, 400 mg L^−1^, and 600 mg L^−1^) were tested against *R. solani* Riz4 colony growth. Each dose was used to amend PDA medium. Agar plates were inoculated with a pathogen plug (6-mm diameter) in the centre and incubated at 20°C. After 3 days, the pathogen colony diameter was measured by a caliber. The sample unit was composed of five plates for each concentration. The control (0 mg/L) was represented by PDA not amended with AP1OD. The assay was conducted twice.

To evaluate the percentage of inhibition of the pathogen colony growth with respect to the control, the following equation was used (Chen and Dai, 2012):


(%) inhibition=(d1−d2)/(d1)×100

where (%) is the percentage of inhibition of *R. solani* colony growth, whereas d1 and d2 control colony diameter (mm) and treated colony diameter (mm), respectively. Finally, the Half maximal Effective concentration (EC_50_) value of the formulation, effective against the pathogen growth, was calculated using the probit model, applied to the percentage of mycelial growth inhibition (Lesaffre and Molenberghs, 1991).

### 2.4 Lettuce seed germination assay

Lettuce seeds (0.01 g corresponding to ∼10 seeds each) were shacked respectively with 10 mL of AP1OD (1 g L^−1^), both SMS extracts (HWE and WWE) and water (control), for 2 h in a rotary shaker (200 rpm). Thereafter, seeds were picked up and distributed on Whatman filters (90-mm diameter) inside sterile Petri dishes and incubated at room temperature. After for 4 days, the perimeter of germinated seeds was measured using Fiji package for ImageJ software, after the image acquisition through a scanner (Epson perfection 2400 photo, Epson, USA).

### 2.5 *In vivo* assays: plant growth promotion and biocontrol effects

Lettuce seedlings, at the fifth true leaf, were transplanted in pots (10 cm × 10 cm × 8 cm) containing 40 g of expanded clay and 120 g of soil (Typ 3, Brill, Germany). To verify the plant growth promotion activity of AP1OD, WWE, and HWE, seedlings roots before being transplanted were submerged 1 h in 25 mL of each treatment (sterile water as control). The treated seedlings were grown at 25°C under greenhouse conditions. After 40 days, five plants per thesis were randomly selected among the 15 cultivated. The plants were carefully extracted and cleaned from the soil to maintain intact the roots and the leaves. The pictures of the selected plants were acquired using a scanner at 2,000 dots per inch _(dpi)_ and analyzed using Fiji package for ImageJ.

To evaluate the antifungal efficacy of AP1OD formulation, 1 g of *R. solani* mycelium was inoculated every 120 g of soil, as reported by Di Francesco et al. (2021). The soil was well mixed and let rest for 24 h. Seedlings, treated as reported above, were transplanted in the pots containing Riz4 mycelium. To evaluate the effects of the formulation against the soil-born pathogen, a disease symptoms index (DSI) was calculated using the following formula (Cardoso and Echandi, 1987):


$$DSI=\frac{percentage\;of\;incidence\;\times mean\;severity}{5}$$

The disease severity (%) was calculated on the basis of a scale from 0 to 4 developed by Khangura et al. (1999) with some modifications, where 0 = no lesions, 1 = lesions covering 1% to 25% of the hypocotyl, 2 = lesions covering 26% to 50% of the hypocotyl, 3 = lesions covering from 51% to 75% of the hypocotyl, and 4 = lesions covering from 76% to 100% of the hypocotyl. Plants transplanted in soil without AP1OD treatment were considered as control. The DSI was calculated on 10 plants after 45 days from pathogen inoculation. The experiments were conducted twice.

### 2.6 Lettuce leave gene expression

Lettuce leaves collected from five plants were sampled 45 days after treatments and stored at −80°C in a pooled fashion. Pooled leaves were grinded in liquid nitrogen and aliquoted in three biological replicates from which total RNA was extracted by Spectrum Plant Total RNA Kit (Sigma-Aldrich). Concentration and quality of RNA samples were verified using the NanoDrop ND-1000 spectrophotometer (ThermoFisher Scientific, Inc., Wilmington, DE, USA), and concentration was adjusted prior to retrotranscription. Two micrograms of total RNA for each sample were processed to obtain a final concentration of 50 ng µL^−1^ of cDNA. The cDNA was reverse-transcribed by means of QuantiTect^®^ Reverse transcription Kit (Qiagen). Extracted cDNA was kept at −20°C until further analysis. A total of four genes of *L. sativa* were selected (**Table 1**) to verify their expression levels in lettuce plants treated with the formulation AP1OD and the SMS extracts (HW and WW). As housekeeping gene, *tip41* gene was selected, as stable in conditions of abiotic stresses, drought, salinity, UV-C irradiation, heavy metals, and with ABA application (Borowski et al., 2014). Gene *ggps* was found differentially expressed in lettuce in correlation to higher antioxidant potential (Damerum et al., 2015), *hppd* gene is involved in vitamin E biosynthesis (Ren et al., 2011; Borowski et al., 2014) and *pdx1* gene in vitamin B6 *de novo* biosynthesis (Chen and Xiong, 2005; González et al., 2007; Sang et al., 2011). Primers for *pdx1* gene were designed by using the online Primer-BLAST design tool on the *L. sativa* genes. Real Time (RT)-PCRs were performed in a final reaction volume of 14 μL per reaction in a 96-well Bio-Rad CFX96 Real-Time PCR System (Bio-Rad Inc., Hercules, CA, USA), in white-walled PCR plates with clear adhesive sealers. Reaction mixtures contained 0.3 μM each primer, 1× SsoFast™ EvaGreen ^®^ Supermix (Bio-Rad Inc., Hercules, CA, USA), molecular grade H_2_O, and 5 ng of cDNA as a template. Cycling conditions were as follows: initial denaturation at 95°C for 30 s; 50 cycles of 5 s at 95°C; and 5 s at 57°C (GGPS f/r, and PDX1 f6/r6) or 60°C (TIP41 f/r and HPPD f/r) depending on the primer pair used. A high-resolution melting curve analysis (ramp from 65°C to 95°C with 0.5°C temperature increments and holding time of 5 s) was programmed at the end of the cycling reaction to evaluate the purity of the amplification product. For each gene, a standard curve on cDNA pooled from all the samples was produced. The pool results were used to set the threshold level for the gene expression analysis on samples to calculate efficiency of the amplification and to obtain the gene expression ratio with the method developed by Pfaffl (2001):

**Table 1:** Primers used for gene expression analysis.

Gene	Primer names	Primer sequences	Primer origin
***tip41***	TIP41 f/r	f-GAGAGATTTGCTGGAGGGAAACTA r-CCTTTGACTGATGATGTTTGGA	Borowski et al. (2014)
***hppd***	HPPD f/r	fCCGGCGCCTTCGTTGTGTTCCAGATAC rGCCCGGGTTTGAACCAGTTGAAAAG	Borowski et al. (2014)
***ggps***	GGPS f/r	f-TTGATTTTTCGATCCCCAAC r-GGCTTTTGTTTCAGGTGGTG	Damerum et al. (2015)
***pdx1***	PDX1 F6/r6	f6-AACAAGCCCGGATAGCAGAA r6-CACCGCCTGAACAATAGCAC	Newly designed on XM_023878089.2 (LOC111881704) by primer BLAST design tool


$$Gene\;\exp ression\;ratio=\frac{{\left(E_{GOI}\right)}^{\Delta CT\;GOI}}{{\left(E_{HKG}\right)}^{\Delta CT\;HKG}}$$

where E_GOI_ = gene of interest standard curve efficiency; E_HKG_ = housekeeping gene standard curve efficiency; DCT GOI = difference in cycle threshold between the gene of interest in the standard curve and in sample tested; and DCT HKG = difference in cycle threshold between the housekeeping gene in the standard curve and the tested sample.

### 2.7 Images acquisition, elaboration, and statistical analyses

All the pictures of the seedlings and the plants were elaborated using Fiji distribution of ImageJ version 1.54 (Schindelin et al., 2012). Each picture contained a ruler as reference for the dimensions.

Data were analyzed by ANOVA one-way analysis and the separation of means was performed with Tukey’s test (α = 0.01 and α = 0.05) by using the software MiniTab.16. Data were reported as mean values ± standard error (SE). The EC_50_ of AP1OD formulation was calculated using the probit analysis applied to the percentage of mycelial colony growth inhibition (Lesaffre and Molenberghs, 1991).

## 3 Results

### 3.1 Chemical analysis of spent mushroom substrate

The chemical characterization of the extracts WWE and HWE obtained respectively at 40°C and 90°C from *A. bisporus* SMS was reported in **Table 2**. No significant differences were determined between the reducing sugar content of WWE (1.22 ± 0.27 g L^−1^) and HWE (1.48 ± 0.30 g L^−1^). Conversely, an effect of the extraction temperature was highlighted on proteins and polysaccharides amount. A higher content of proteins and polysaccharydes was detected in the WWE, respectively 857.56 ± 8.91 mg L^−1^ and 1,199.44 ± 9.79 mg L^−1^. The extraction at mild conditions allowed an increase of proteins and polysaccharides, respectively, by 7% and 25%, when compared to the HWE. SE-HPLC analysis distinguished two polysaccharides’ fractions based on their molecular weight estimation. The warm water allowed to extract polysaccharides fractions with a molecular weight 30% and 15% smaller than the HW extraction. The extraction temperature affected not only the solutes content but also their chemical characteristics.

**Table 2:** Chemical composition of *Agaricus bisporus* (Ab) spent mushroom substrate (SMS) WWE (40°C) and HWE (90°C).

		WWE SMS Ab	HWE SMS Ab
Extraction temperature		40°C	90°C
***Reducing sugars***	(g L^−1^)	1.22 ± 0.27 a^*^	1.48 ± 0.30 a
***Proteins***	(mg L^−1^)	857.56 ± 8.91 b	800.42 ± 21.69 a
Polisaccharides			
Total content	(mg L^−1^)	1199.44 ± 9.79 b	901.50 ± 48.58 a
Compound 1			
Content	(mg L^−1^)	336.51 ± 16.12 b	258.55 ± 22.46 a
Molecular weight	(kDa)	1.25·10^7^	1.75·10^7^
Compound 2			
Content	(mg L^−1^)	862.93 ± 6.80 b	642.95 ± 30.01 a
Molecular weight	(kDa)	1.03·10^4^	1.21·10^4^

*Each data represents the mean of three replicates ± standard deviation.

Different letters within line indicate significant differences (α < 0.05).

### 3.2 *In vitro* assay: efficacy of AP1OD formulation against *R. solani* mycelial growth and EC_50_ value

The formulation was tested to assess the effectiveness of the yeast strain AP1 following the formulation process. Pathogen colony growth (Ø, mm) was evaluated after 3 days of incubation at 20°C (**Table 3**). Exclusively to the highest tested concentrations (600 mg L^−1^ and 400 mg L^−1^), AP1OD formulation displayed an inhibition on average by 57% with respect to the control in inhibiting *R. solani* Riz4 mycelial growth. Conversely, at 50 mg L^−1^ and 100 mg L^−1^, a slight pathogen colony growth promotion was observed. The mycelial inhibition growth data were used to calculate the AP1OD formulation EC_50_ value that corresponded to 476.29 mg L^−1^ (data not shown).

**Table 3:** Inhibitory effect (A) of AP1OD formulation amended with PDA in five different concentrations, ranging between 0 mg L^−1^ (control) and 600 mg L^−1^.

AP1OD (mg L^−1^)	Colony diameter (mm)
600	22.25 ± 1.39 a
400	20.38 ± 1.60 a
200	48.33 ±1.50 b
100	55.50 ± 0.76 c
50	53.75 ± 1.75 c
0	49.33 ± 1.51 b

Data are expressed as colony diameter of Riz4. Different letters indicate significant differences according to Tukey’s test (α = 0.05).

### 3.3 *Lactuca sativa* seed germination

**Figure 1:**
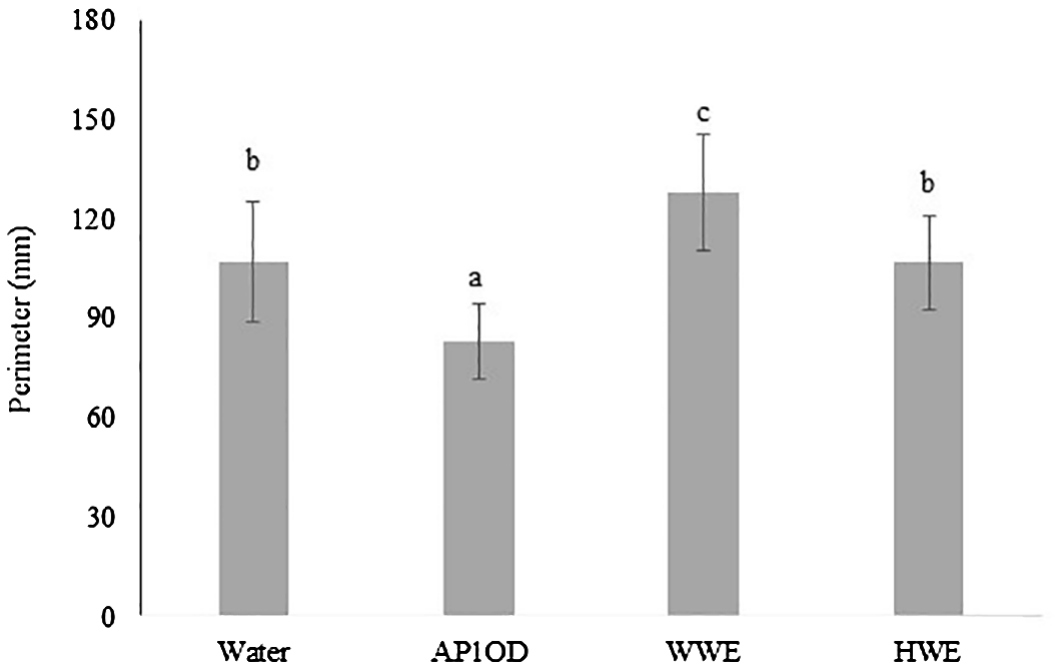
Perimeter of germinated lettuce seeds (10 mg) previously treated with AP1OD formulation (1 g L^−1^, 10 mL) and SMS extracts of *Agaricus Bisporus* (WWE and HWE, 10 mL). Sterile tap water was used as control (10 mL). Treated seeds were incubated in a Petri plate for 4 days at room temperature. Data are the mean of 10 sprouted seeds ± standard deviation measured using Fiji ImageJ version 1.54 (Schindelin et al., 2012). Different letters indicate significant differences according to Tukey’s test (α = 0.05).

The effect of AP1OD and *A. bisporus* SMS on lettuce seed germination was evaluated after for 4 days from the treatments. The image of germinated seeds was acquired through a scanner and the perimeter (mm) of treated germinated seeds was measured by an image software. Germinated seeds’ perimeter measurements were reported on **Figure 1** and supported by **Supplementary Figure 1**. Only the treatment with SMS extract obtained by warm extraction showed an increase of the seedling perimeter by 27%, compared to the control. On the other hand, HWE do not affect the seedlings perimeter with respect to the water control. The formulation AP1OD negatively affected the lettuce seed germination determining a significant decrease by 22%, compared to the control.

### 3.4 *In vivo* assays

**Figure 2:**
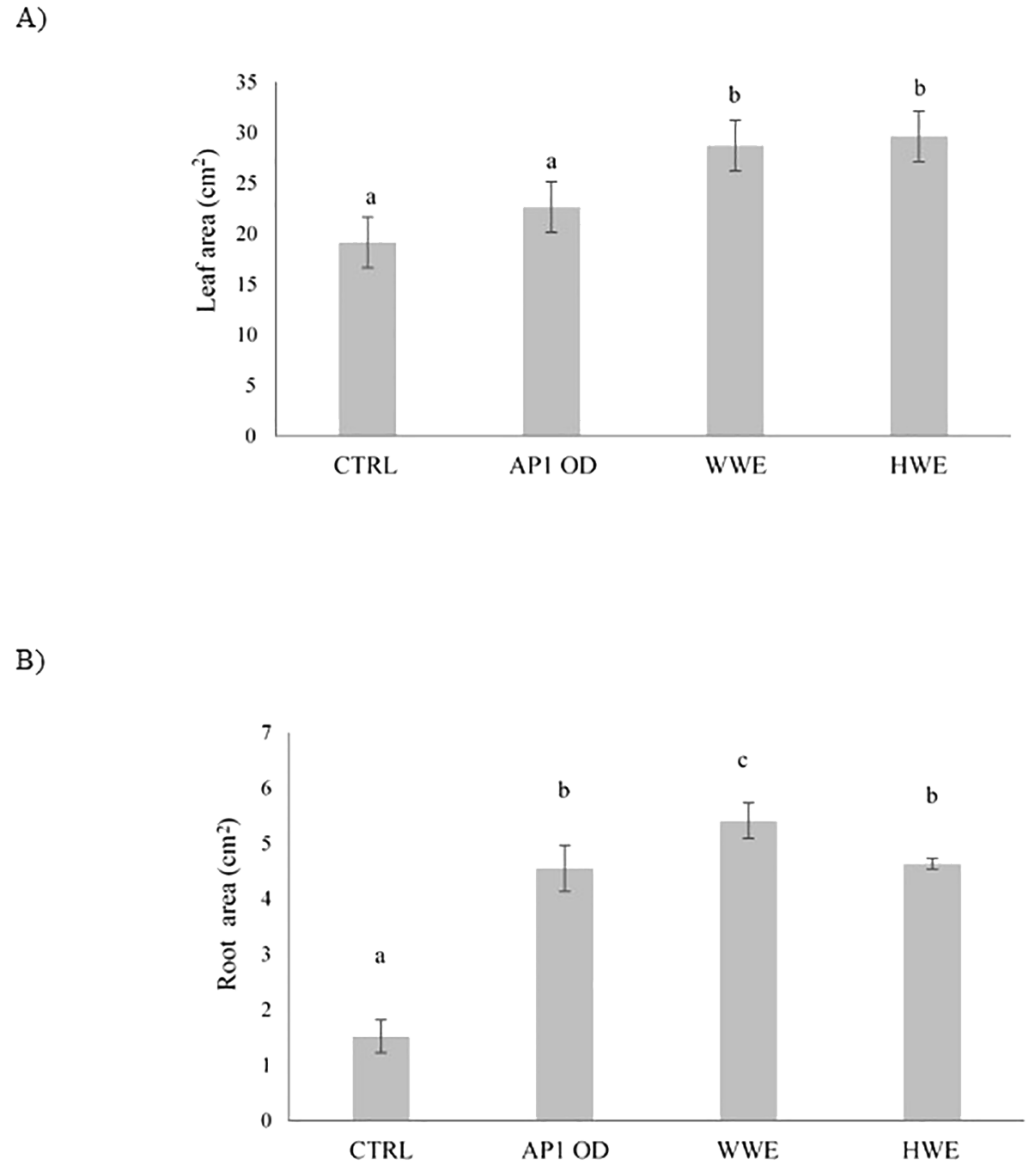
Leaves **(A)** and root area (cm^2^) **(B)** of treated and water treated (control) lettuce plants ± standard deviation. Plants were treated with AP1OD formulation and *Agaricus bisporus* SMS extracts (WWE and HWE). Data are the mean of five plants. Area measurements were obtained using Fiji ImageJ version 1.54 (Schindelin et al., 2012). Different letters indicate significant differences according to Tukey’s test (α = 0.01).

After 40 days, lettuce plants previously treated with AP1OD formulation (1 × 10^7^ cells g^−1^) and SMS extracts (WWE and HWE) were analyzed on the bases of leaves (**Figure 2A**) and roots area (**Figure 2B**). Regarding the lettuce leaves area, both *A. bisporus* SMS extracts (WWE and HWE) increased the leaves surface by 50% compared to the control. AP1OD showed no substantial effects. Conversely, all the treatments, especially WWE, stimulated the roots growth, displaying an increase on average higher than 200% if compared to the control. Data were supported by **Supplementary Figure 2**.

**Figure 3:**
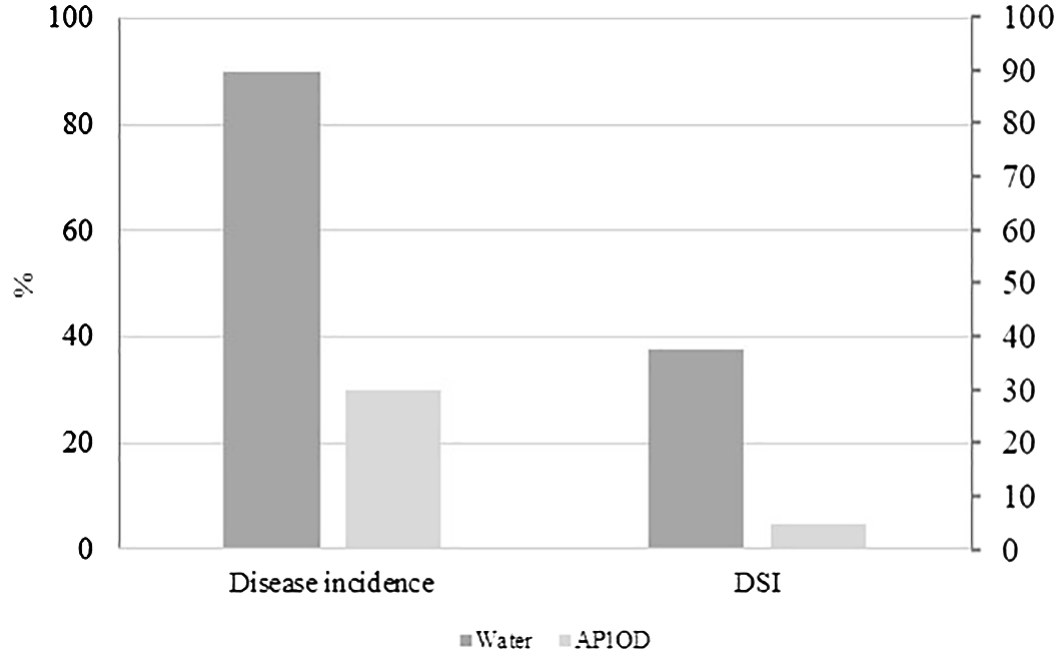
Efficacy of AP1OD formulation against *Rhizoctonia solani* symptoms in lettuce plants. The figure reported the percentage of disease incidence (%) of treated (1 g L^−1^ of AP1OD) and untreated plants (water) and the relative disease severity index (DSI) verified after 40 days after the artificial pathogen inoculation.

Regarding the biocontrol assay, AP1OD formulation used against *R. solani* (Riz4) reduced by 66.6% the soil-borne pathogen incidence, corresponding to the disease severity index (DSI) of 4.8. The water control displayed a DSI of 37.8 (**Figure 3**).

### 3.5 Gene expression

**Figure 4:**
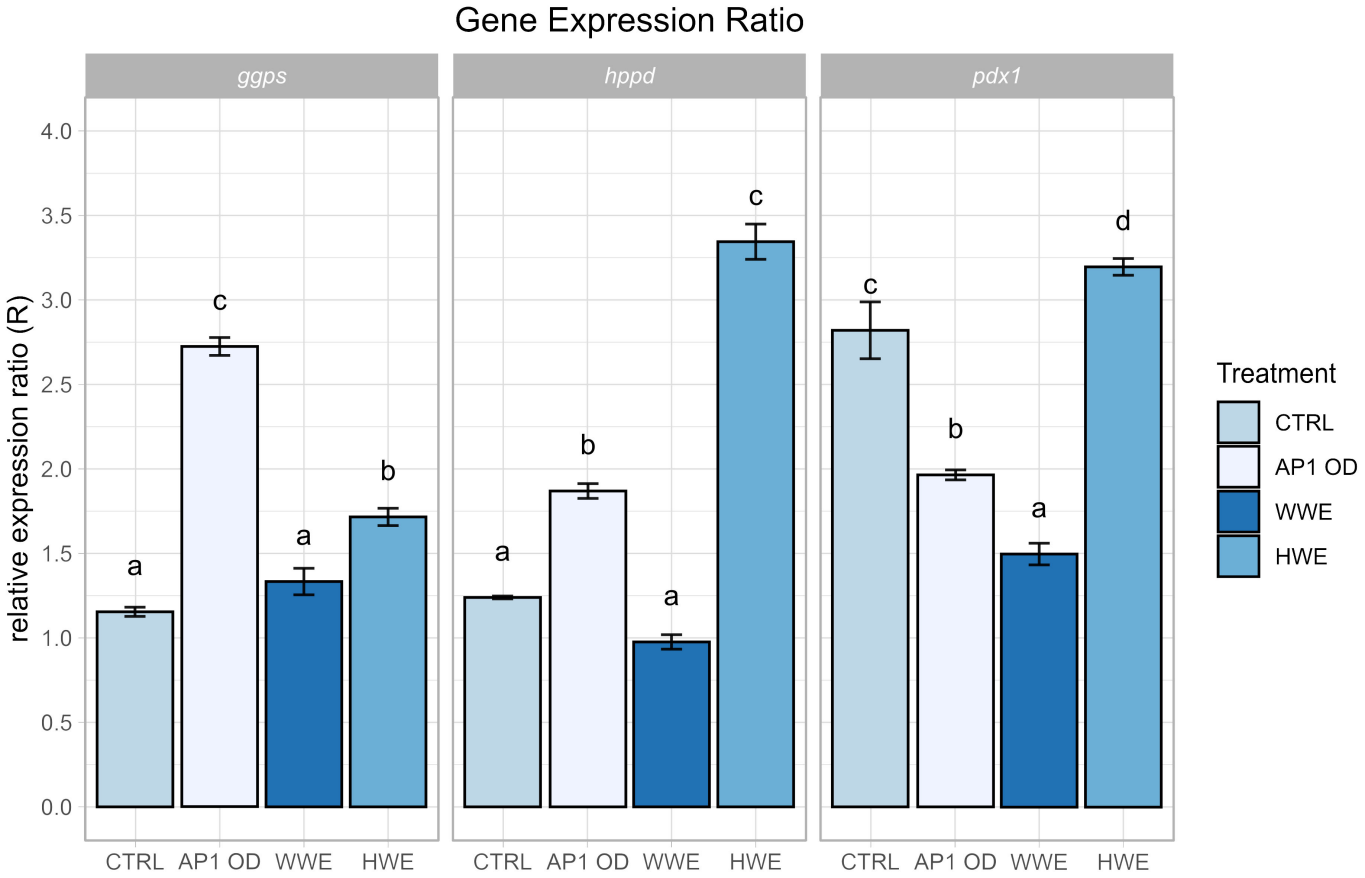
Gene expression ratios for AP1OD, WWE, and HWE *Agaricus bisporus* SMS extracts treatments on *ggps*, *hppd*, and *pdx1* genes. The data reported are the mean of three replicates ± standard error. Different letters indicate significant differences according to Tukey’s test (α = 0.05).

By the gene expression analysis (**Figure 4**), *ggps*, *hppd*, and *pdx1* genes evidenced how AP1OD formulation and HWE treatments increased their expression levels. The WWE was the only treatment which did not show an increasing of genes expression level with respect to the control for all the three genes. In case of *ggps*, gene involved in the regulation of antioxidant biosynthesis, both treatments, AP1OD and HWE, determined an increase of expression by 1.36 and 0.49 times, respectively. Following the same trend, the expression of *hppd* gene, involved in vitamin E biosynthesis, was enhanced by 0.51 and 1.70 times in relation to AP1OD and HWE treatments. On the other hand, AP1OD formulation not stimulated an increase in the *pdx1* expression level as above reported for the other genes, compared to the control. The treatment with HWE confirmed its influence in increasing by 0.13 times the *pdx1* gene in lettuce with respect to the untreated plants.

## 4 Discussion

In the present study, two different organic approaches for lettuce crop management were considered, the first consisted of AP1OD, an experimental formulation of *A. pullulans* AP1 strain, and the second of bio-extracts derived from SMS of *A. bisporus*, obtained by two different extraction temperature.

Although *Aureobasidium* spp. are microorganisms mainly known to be a BCA active against many postharvest fungal pathogens (Di Francesco et al., 2020), the present study evaluated its ability to control a soil-born pathogen and also its plant biostimulation ability. However, it is one of the most frequently genera of soil yeasts together with *Candida* spp., *Cryptococcus* spp., and *Rhodotorula* spp (Spencer and Spencer, 1997). The prototype of formulation AP1OD already showed a good performance against *Botrytis cinerea* of table grape (Cignola et al., 2024). Cignola et al. (2024) showed that OD formulation ensured a great cells viability overtime, probably due to the oil protective action against oxidative stresses and water exchanges. Confirming that, by *in vivo* assay, the efficacy of AP1OD formulation against the soil-borne pathogen *R. solani* Riz4 was displayed, validating that the use of BCAs could represent a suitable strategy to control fungal pathogens (El-Tarabily, 2004; Grosch et al., 2012; Di Francesco et al., 2021; Cignola et al., 2024). Recently, in the agricultural sector, the use of BCAs was also considered for the plant growth promotion activity (Palmieri et al., 2022). Because of this, the ability of the formulation AP1OD was also verified as a biostimulant on the bases of lettuce leaf and root area dimensions. Contrary to that demonstrated by Patkowska (2021), the tested formulation AP1OD showed a better efficacy in countering the fungal pathogen growth with respect to the biostimulation capability. In fact, the technological properties of a bioformulation are very important requirements for the exploitation of the active substance (BCA) mechanisms of action. In this case, OD guarantees an increased persistence on the host surface (Mbarga et al., 2014) and also on the lettuce roots. Probably, yeast roots colonization (Di Francesco et al., 2021; Palmieri et al., 2022) ability, together with the production of secondary metabolites (Di Francesco et al., 2021) could explain the reduced damages caused by *R. solani*.

About SMS extracts, HWE and WWE used as soil amendments induced an increase of lettuce leaves and roots area, confirming their high potential for greenhouse cultivations (Martín et al., 2023).

Because SMS constitutes a significant portion of the overall waste in mushroom production, the use of its extract as PGP would reduce the amount of generated waste. To date, common practice to deal with the byproduct is a pasteurization at 60°C before composting (Rocha Vieira and Pecchia, 2021). The treatment proposed for the extraction might substitute the pasteurization process while still leaving material for composting. The temperature has a significant effect on extraction efficiency and it can modify properties of bio-extracts. The increase of temperature allows an increase of solutes’ solubility and diffusivity but can induce the degradation of thermosensitive compounds that would decrease the extract quality (Barbero et al., 2022; Priego-Capote, 2021).

The SMS extraction at the lowest temperature (40°C) showed the best results and avoided thermal degradation of the solutes. The increase of temperature at 90°C could trigger solutes thermal degradation or other chemical process, such as protein denaturation and Maillard reaction, with a significant decrease of proteins and polysaccharides content (De Oliveira et al., 2016; Dursun Capar and Yalcin, 2021). Moreover, the increase of temperature showed a significant effect on polysaccharides fractions and their molecular weights (**Table 2**). Higher extraction temperature increases the solubility and the diffusion of bigger macromolecular substances in water, and it could influence the polysaccharides conformation and composition (Nurmamat et al., 2018).

The analyzed lettuce genes, *hppd* and *pdx1*, that are involved in vitamin biosynthesis widely resulted in expression increase after the plant treatment with both AP1OD and HWE, confirming the potential of the carried-out treatments. Specifically, *hppd* gene catalyzes the initial steps of vitamin E biosynthesis, leading to the production of α-tocopherol and plastoquinone (Mène-Saffrané, 2017). The existing literature consistently demonstrates a positive correlation between *hppd* gene expression and vitamin E content (Jiang et al., 2017; Mène-Saffrané, 2017; Kim et al., 2021). Therefore, the rise in expression of *hppd* for AP1OD and HWE treatments could signify an increase in vitamin E content.

The increasing content of vitamin E may be interpreted as a strategy for plants to reduce stress and provide a better antioxidant protection (Holländer-Czytko et al., 2005; Lushchak and Semchuk, 2012). Also, as reported by Mene-Saffranè et al. (2010), plants lacking tocopherols exhibit greater lipid oxidation and poor seed germination and substantially shorter roots at maturity, so indicating their importance for plant growth and survival.

In contrast with vitamin E, vitamin B6 biosynthesis metabolism is constituted of both a *de novo* pathway, mediated by *pdx* enzymes (Fitzpatrick, 2011). In lettuce plants treated with HWE, an increment in *pdx1* gene expression was detected. It could be translating as a higher content of B6 vitamin with respect to the lettuce treated with AP1OD and WWE.

Among the investigated genes, geranylgeranyl pyrophosphate synthase (*ggps*) codifies for an enzyme involved in terpenoid biosynthesis that was found to be differentially expressed in lettuce in correlation with antioxidant potential (Damerum et al., 2015). Also, geranylgeranyl pyrophosphate (*ggpp*) is an intermediate in the biosynthesis in plants of essential compounds as chlorophyll, carotenoids, tocopherols, phytoalexins, plastoquinones, and gibberellins (Rodríguez-Concepción and Boronat, 2002). The involvement of terpenes in plant resistance to fungal diseases has been previously demonstrated, as reported by Toffolatti et al. (2021). Increase in *ggps* expression in plants treated with AP1OD and HWE could, thus, reflect an increase of antioxidant potential as well as an enhancement of defenses against biotic and abiotic stresses (Damerum et al., 2015).

Concluding, due to the present study, the application and efficacy of a new formulation prototype based on a*. pullulans* strain and two by-products derived from SMS were evaluated against the soil-borne pathogen *R. solani*. The prototype showed a considerable control of the target soil-borne pathogen, and, at the same time, the potential of mushroom by-products as PGPs and nutraceutical value-added enhancers was considered. The proposed solutions may represent possible alternatives to plant management, both from phytopathological and agronomic perspectives, that look carefully at new sustainable possibilities to adopt in agricultural systems.

However, these results need further chemical investigation to analyze the vitamin levels in the treated plants as well as the simultaneous use of the two treatments (formulation and SMS extracts) to verify the possibility of a complementary mode of action in inhibiting the pathogen and stimulating the plant growth.

## Data Availability

The raw data supporting the conclusions of this article will be made available by the authors, without undue reservation.
