# Supplementary material for: Effects of bioformulation prototype and bioactive extracts from Agaricus bisporus spent mushroom substrate on controlling Rhizoctonia solani of Lactuca sativa L
Source: Front Plant Sci. 2024 Oct 24;15:1466956. doi: 10.3389/fpls.2024.1466956 (PMC11540695; doi:10.3389/fpls.2024.1466956)

**Supplementary material S1.** Lettuce seeds after 4 days of incubation at room temperature, treated with AP1OD formulation (1 g L^-1^) (B) and SMS extracts of *Agaricus bisporus* WWE (C) and HWE (D), 10 mg of seeds were wetted with 10 mL of the different treatments. Sterile water was used as control (A).
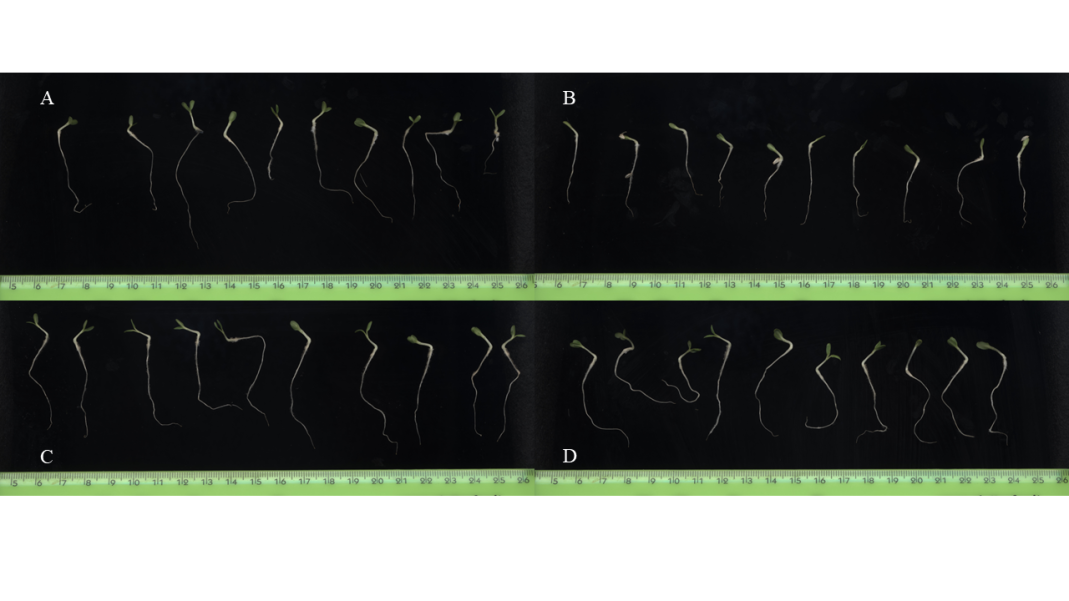

Supplement: Supplementary file 1 [file Table1.docx]
