# Supplementary material for: Effects of bioformulation prototype and bioactive extracts from Agaricus bisporus spent mushroom substrate on controlling Rhizoctonia solani of Lactuca sativa L
Source: Front Plant Sci. 2024 Oct 24;15:1466956. doi: 10.3389/fpls.2024.1466956 (PMC11540695; doi:10.3389/fpls.2024.1466956)

**Supplementary material S2.** Leaves (1) and roots (2) of lettuce plants treated with water (A), AP1OD formulation (B) and *Agaricus bisporus* WWE (C) and HWE (D).

1)


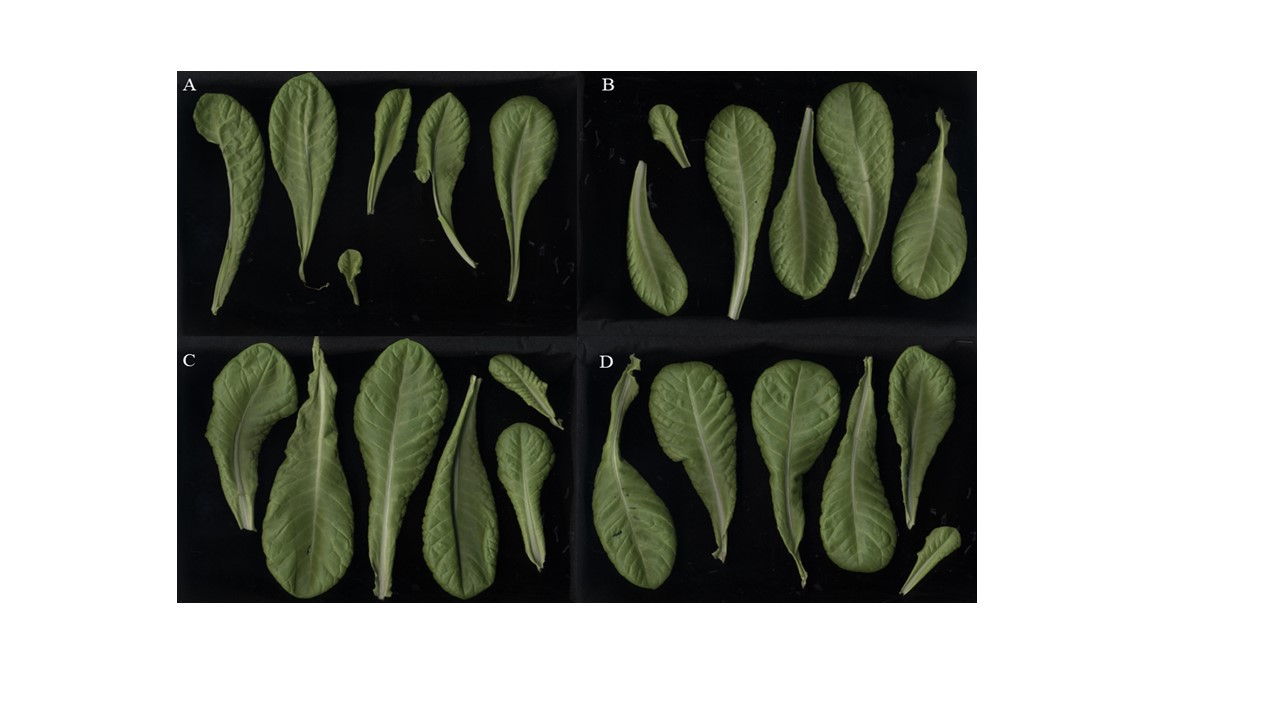


2)


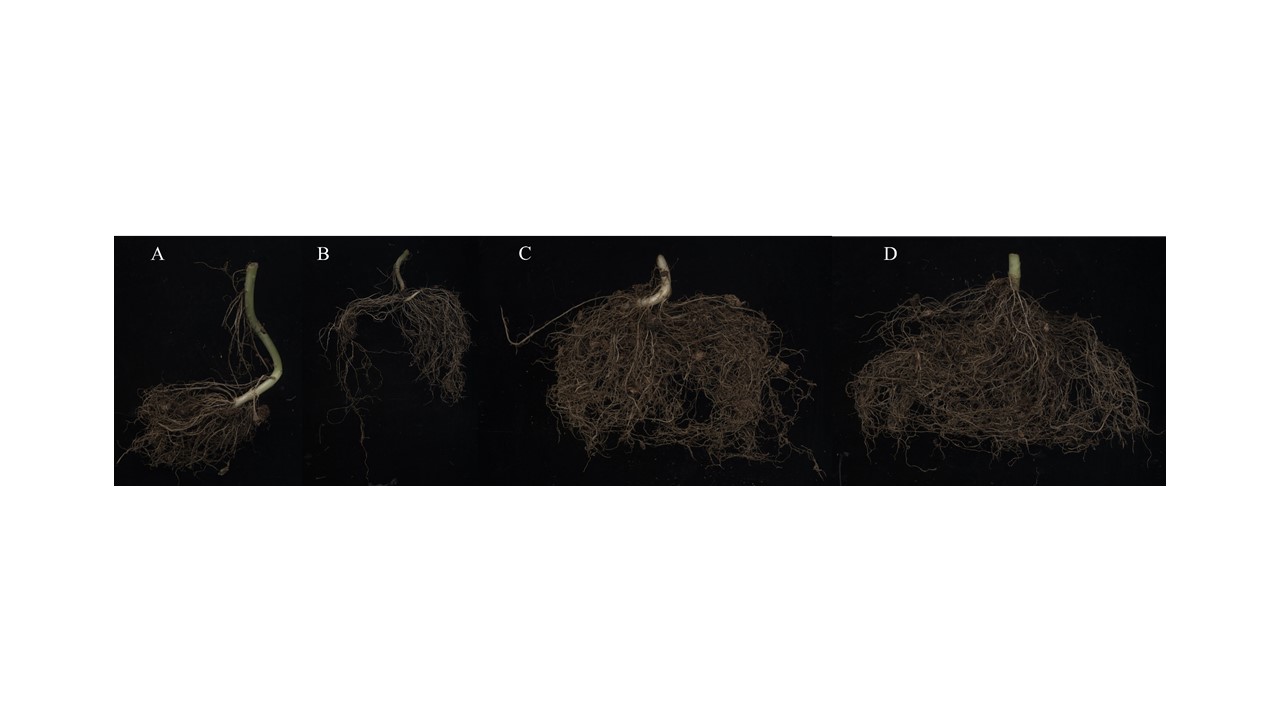

Supplement: Supplementary file 2 [file Table2.docx]
